# Calciphylaxis in a 30-Year-Old Woman With Alcoholic Cirrhosis: A Case Report

**DOI:** 10.7759/cureus.69527

**Published:** 2024-09-16

**Authors:** Adrian Parra, Russ A Kuker

**Affiliations:** 1 Department of Radiology, University of Miami Miller School of Medicine, Miami, USA; 2 Department of Radiology and Nuclear Medicine, Jackson Memorial Hospital, Miami, USA

**Keywords:** nuclear bone scan, liver cirrhosis, end stage renal disease (esrd), bone scintigraphy, non-uremic calciphylaxis

## Abstract

A diagnosis of calciphylaxis is rare amongst the vulnerable population of patients with end-stage renal disease (ESRD); however, it has poor outcomes when it does present. With ineffective clearance due to reduced kidney function, calcium and phosphorus accumulate and deposit in the intimal layer of blood vessels and other soft tissues throughout the body. It can be proven using biopsy of skin lesions characteristic of the disease or with less invasive methods including X-ray and bone scintigraphy. Calciphylaxis is typically seen in middle-aged patients who have undergone prolonged dialysis treatment and has a devastating prognosis unless the patient can obtain a renal transplant. In this report, we present a case of a 30-year-old female patient with calciphylaxis and highlight the value of bone scintigraphy for diagnosis, while noting the importance of organ transplant for proper treatment.

## Introduction

Calciphylaxis, also known as calcific uremic arteriolopathy, is most often found in older patients with chronic kidney disease (CKD) who have undergone dialysis for an extended period of time, according to a study by Rick et al. [1]. With an inadequate filtration system, calcium, phosphorous, and other minerals accumulate in patients and deposit along their blood vessels, causing insufficient perfusion of their skin, which can ultimately lead to necrosis, as described by Chang [2]. Early identification of this complication is vital in the prognosis of patients and adds a sense of urgency in their treatment plan as mortality rates approach 30% at six months [3]. Providers should be familiar with clinical findings or objective data suggestive of this disease to avoid delays in treatment. However, atypical presentations of calciphylaxis can occur which can make this diagnosis challenging, such as the development in a young patient. There are also instances where patients’ disease has progressed too far before being detected, which necessitate alternative methods of treatment that provide comfort care versus aggressive measures. We present a case of a 30-year-old female with end-stage renal disease (ESRD) who developed calciphylaxis and was unfit to be a transplant candidate, electing to undergo hospice care.

## Case presentation

The patient was a 30-year-old female with a past medical history of metabolic dysfunction associated with steatotic liver disease (MetALD), childhood asthma, and hepatorenal syndrome with renal failure who presented from an outside hospital. She presented with complaints of worsening abdominal distension, lower extremity edema, elevated liver enzymes, and metabolic acidosis.

The patient was hospitalized a month prior to this admission due to altered mental status, was diagnosed with cirrhosis at that time, and was treated for hepatic encephalopathy. She underwent a paracentesis for ascites and was treated for renal dysfunction. Ultimately, she was discharged on lactulose and diuretics. After leaving the hospital, she received frequent outpatient paracenteses.

She subsequently presented to the emergency room again with similar symptoms of shortness of breath and abdominal distension, and her vitals were notable for a blood pressure of 94/37. The patient was noted to have bilateral lower extremity edema, and a lesion on her right shin that was initially thought to be cellulitis. The patient was found to have an acute kidney injury, non-anion gap metabolic acidosis, and elevated lactate level, with specific lab values listed in Table 1. She began to receive hemodialysis with ultrafiltration and was evaluated for a possible liver-kidney transplant, with a Model for End-Stage Liver Disease (MELD) score of 33. Her hospital stay was complicated by multiple transfers to the ICU for episodes of undifferentiated shock requiring vasopressors, metabolic encephalopathy, renal failure, and desaturations requiring ventilatory support.

**Table 1:** Patient lab values with reference ranges and units BUN: blood urea nitrogen; Cr: creatinine; pCO_2_: partial pressure of carbon dioxide; HCO_3_: bicarbonate

Parameter	Result	Reference Range/Normal Values	Units
BUN	75	7-20	mg/dL
Cr	9.3	0.6-1.2	mg/dL
pH	7.3	7.35-7.45	-
pCO_2_	31	35-45	mmHg
HCO_3_	15	22-28	mEq/L
Lactate	2.5	0.5-2.2	mmol/L

**Figure 1:**
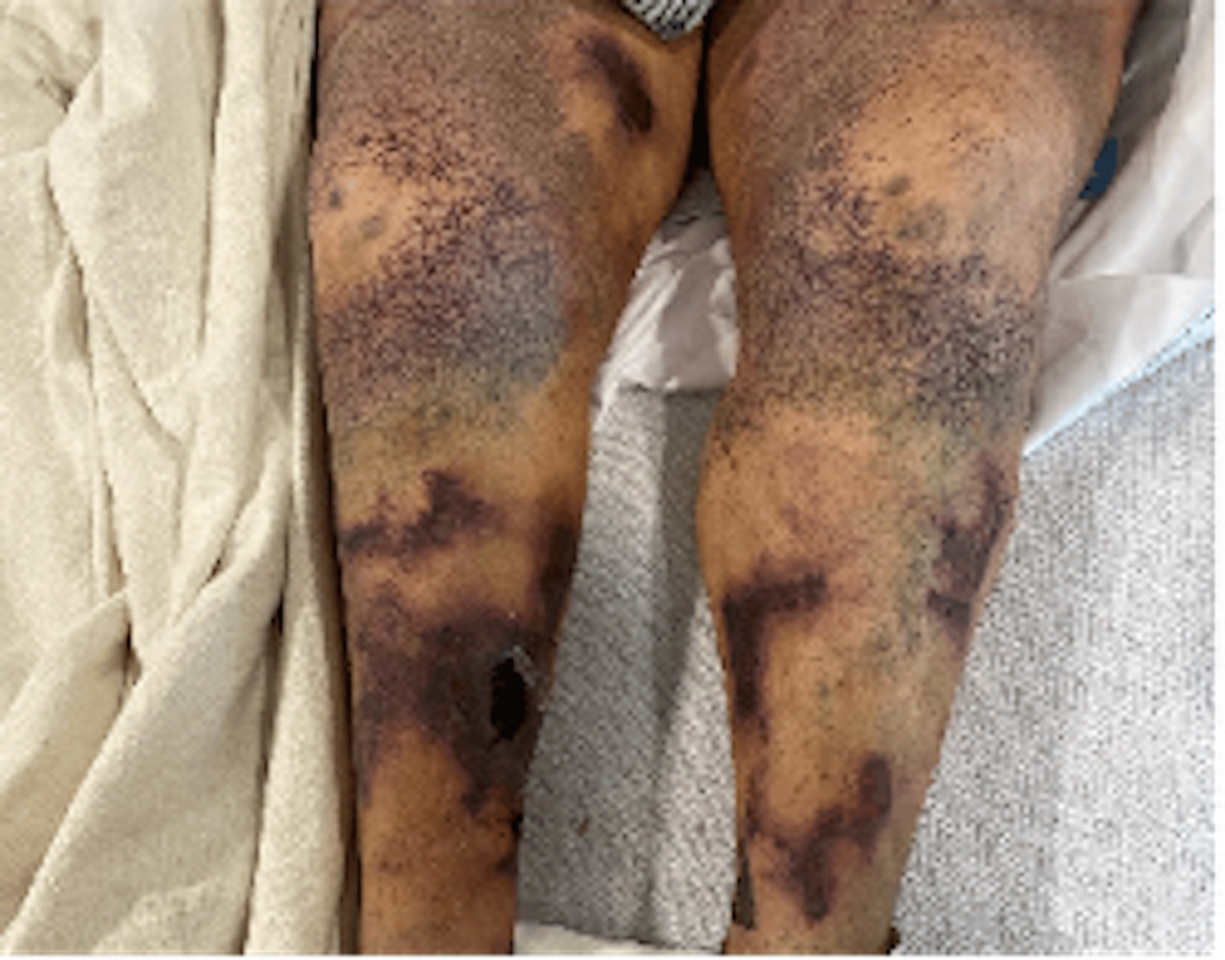
Necrotic ulcers resulting from tissue ischemia due to calcified vasculature and retiform purpura, a classic cutaneous manifestation of calciphylaxis.

After about one month of receiving hemodialysis on a Monday, Wednesday, and Friday schedule during her admission, the patient developed tender petechial rashes on her bilateral lower extremities and chest which was classified as retiform purpura. The lesion on her right shin was now a necrotic eschar, seen in Figure 1. A skin biopsy was taken from the right shin for H&E stain, direct immunofluorescence, and culture. Pathology revealed a large blood vessel in the subcutis with calcification, indicating a vasculopathy and ruling out an infectious process. Deeper sections with von Kossa stain were negative, making a diagnosis of calciphylaxis less likely. The differential was broad and included cholesterol emboli syndrome, vasculitis, antiphospholipid syndrome, and protein C and S deficiency, among others. A panel of labs including antineutrophil cytoplasmic antibodies (ANCA), anti-phospholipid panel, anti-platelet factor 4, factor V Leiden, methylenetetrahydrofolate reductase, proteins C and S, and ADAMTS13 were ordered and came back negative. Parathyroid hormone (PTH) was elevated to 254, along with elevated phosphate levels at 7.2 mg/dL, above the normal range of 2.5 to 4.5.

**Figure 2:**
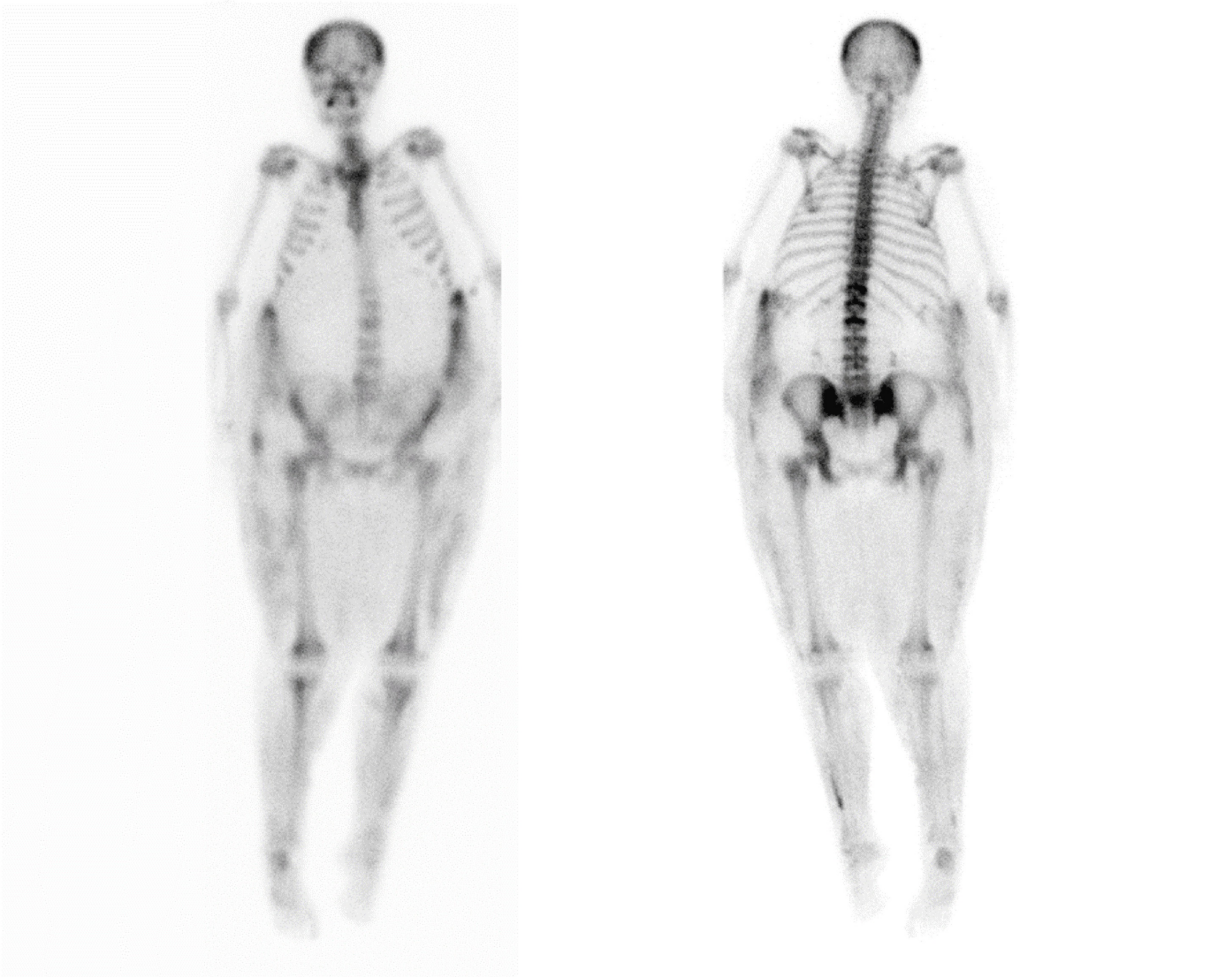
The Tc-99m methylene diphosphonate (MDP) bone scan showed increased radiotracer uptake in the subcutaneous soft tissue at bilateral flanks, bilateral thighs, and bilateral lower legs. There was diffuse, uniform increased radiotracer uptake in the axial and appendicular skeleton including the skull. There is no visualization of the kidneys, which could represent a metabolic SuperScan.

A nuclear medicine whole-body bone scan, seen in Figure 2, was performed with technetium-99m-methylene diphosphonate (MDP) and revealed increased radiotracer uptake in the subcutaneous soft tissue at the bilateral flanks, bilateral thighs, and bilateral lower legs, consistent with calciphylaxis. There was also diffuse uniform increased radiotracer uptake in the axial and appendicular skeleton including the skull which could represent a metabolic SuperScan secondary to renal osteodystrophy which results in secondary hyperparathyroidism as seen in this patient.

The patient was started on sodium thiosulfate with dialysis three times a week for calciphylaxis, along with sevelamer to treat hyperphosphatemia. She was deemed to not be a candidate for transplant due to severe calciphylaxis and was recommended to receive palliative care. After several discussions, the patient agreed to a “Do Not Resuscitate” (DNR) order and a "Do Not Intubate" (DNI) order and received hospice care at home.

## Discussion

Timely treatment of patients with calciphylaxis is imperative, thus making it important to have an accurate and timely diagnosis. Providers have historically relied on the patient's clinical presentations to recommend biopsy [3], and the final diagnosis is established upon the pathological report. This then necessitates consultation with various specialities to obtain a cutaneous specimen, similarly seen in this case when the dermatology team was consulted. A substantial amount of time can be elapsed from the patient's symptom onset to tissue sampling.

Even once these samples are taken, equivocal results are not uncommon when obtaining biopsies from dermatologic lesions secondary to calciphylaxis. Many times, the inflammatory and necrotic debris from the various stages of healing ulcers in these patients make it difficult for pathologists to make an accurate diagnosis as described in an article by Jiménez-Gallo et al. [4], which was what occurred in this specific case.

Utilizing the nuclear medicine study to identify calcium depositions throughout the body is a non-invasive and expeditious method to diagnose calciphylaxis. The underlying mechanism of nuclear medicine bone scans is MDP adsorbs onto the hydroxyapatite crystals in proportion to the osteoblastic activity. It can also deposit in extraosseous soft tissue secondary to calcium deposition, inflammation, infection, or trauma, as described in an article by Itani et al. [5]. According to a *JAMA Dermatology* article [6], bone scintigraphy had an 89% sensitivity and 97% specificity in identifying cases of calciphylaxis. This method not only is accurate in diagnosis, but it also decreases the risk of post-biopsy complications, ranging from bleeding to increasing areas of necrosis. Plain film soft-tissue radiographs are yet another imaging technique that could be used in assessing for cases of calciphylaxis, however, the accuracy of this methodology does not reach the standards of bone scans. A peer-reviewed abstract published by the Radiological Society of North America (RSNA) [7] demonstrated that X-ray has a specificity of 90% in identifying calciphylaxis.

The value of bone scan in patients at risk for this disease is not only for diagnosis and treatment, but is also vital for prognosis. This imaging technique can provide patients and physicians with an idea of the extent of disease as well as for choosing the appropriate treatment for the patient. As seen in this unfortunate case, the patient’s extent of disease was past the point of renal transplantation.

## Conclusions

An accurate and timely diagnosis of calciphylaxis is detrimental to the patient care of this disease. This calciphylaxis case demonstrated complicated clinical signs and symptoms and underwent extensive workup including laboratory studies, tissue biopsy, as well as nuclear medicine bone scintigraphy. Providers must be aware of the non-invasive character and effectiveness of nuclear medicine bone scintigraphy in the diagnosis and prognosis of this disease, despite dermatologic biopsy being the gold standard.
